# Exploring Jordanian nurses’ attitudes toward conducting nursing research: cross-sectional study from Jordan

**DOI:** 10.3389/fmed.2025.1646739

**Published:** 2025-07-30

**Authors:** Ahmad Hussein Al-Duhoun, Raya Y. Alhusban, Fatimah S. Tarawneh, Kamlah A. Al-Olaimat, Maha Atout

**Affiliations:** ^1^Faculty of Nursing, Mutah University, Karak, Jordan; ^2^Faculty of Nursing, Zarqa University, Zarqa, Jordan; ^3^Princess Muna College of Nursing, Mutah University, Karak, Jordan; ^4^Department of Obstetrics and Gynecologic Nursing, Faculty of Nursing, Zarqa University, Zarqa, Jordan; ^5^Faculty of Nursing, Philadelphia University, Amman, Jordan

**Keywords:** nursing research, attitude, conducting research, Jordan, cross-sectional study

## Abstract

**Background:**

Research is essential in nursing to augment the existing knowledge foundation underpinning the field, from education through practice and administration. To date, few studies have explored this topic in Jordan or the broader Middle Eastern region, and this study addresses this important gap.

**Objective:**

The objective of the current study was thus to examine the perspectives of Jordanian nurses concerning the practice of nursing research.

**Methods:**

In a cross-sectional descriptive study, 220 nurses from public, private, and academic institutions in Jordan were approached using a convenience sampling to complete the Boothe Attitudes Toward Nursing Research Scale.

**Results:**

The results showed that the overall mean score of all Boothe subscales was 3.289, SD = 0.968, reflecting practitioners’ uncertainty as a cohort in their attitudes toward research. Nurses exhibited somewhat favorable attitudes regarding the incentives and benefits associated with conducting nursing research, however (mean = 3.439, SD = 1.038). Statistically significant effects were also seen for participants’ level of education (*F* (2,218) = 39.993, *p* < 0.000), their personal interest in research (*F* (2,218) = 26.251, *p* < 0.000), and whether they received an incentive for doing studies.

**Conclusion:**

This study suggests that if hospital administrators work to collaborate with the academic institution to advance and facilitate higher education, they can improve patient outcomes by ensuring that more nursing practices are safe and evidence-based.

## Background

Scientific research is essential for all disciplines, and it is crucial for delivering effective and safe care within health care services. Nursing is the primary and most diverse field within the healthcare profession, and research is thus vital to nursing with respect to enhancing the underlying knowledge base supporting practice, education, and administration in this field (1). Research in this context refers to systematic and organized inquiry into a specific phenomenon to acquire facts and results (2). The term “research” can thus refer to processes of both re-examination and meticulous investigation, encompassing a broad range of activities related to collecting, analyzing, and applying information (2).

Ongoing research is essential for a good healthcare system, aiming to explore unknown areas of reality using data, statistics, and observations to help both healthcare workers and the public, and nursing has greatly increased its knowledge through this research over time (2). Research is also undertaken as a partial requirement for most academic degrees in nursing, and consequently, research utilization and implementation have intensified as these nurses deliver high-quality nursing care to patients. Nurses at various educational levels and specializations should be encouraged to participate in and conduct research to achieve this outcome, though numerous nursing journals have also emerged to disseminate studies relevant to nurses. In this way, nursing increasingly relies on evidence-based practice, leading to a need to incentivize nurses to engage in research and to ensure no impediments to their efforts.

According to Devrani et al. (3), however, a disparity exists between nurses’ attitudes and their actual research practices. While 84% of nurses indicated a favorable disposition toward research, merely 14% delivered presentations at conferences, and 10% published articles in academic journals. Similarly, Ünver et al. (4) discovered that while the majority of nursing students felt positive about scientific research, only 13.6% of them had conducted it.

Numerous studies have also identified obstacles that may impede nurses from engaging in research, including organizational barriers (5, 6), difficulties in comprehending research concepts (7), and a lack of confidence in conducting research (6). Numerous studies have thus investigated the demographic characteristics of nurses, including educational attainment, age, experience, and job role, to determine any associations between these factors and attitudes toward nursing research (3, 8). Some studies have identified a correlation between such attitudes and education level, age, and years of research experience (8). In contrast, other studies have found no significant relationship between nurses’ professional qualifications (3) or age in terms of nurses’ attitudes toward nursing research (7). Heydari and Zeydi (9) reviewed eleven papers with the aim of identifying obstacles and enablers of research utilization among Iranian nurses; they determined that a lack of administrative support, insufficient time, and absence of power impeded nursing research utilization. In contrast, education on research provided by proficient colleagues and nursing faculty members, access to financial resources, and proficiency in English were enablers for the application of nursing research (9).

In Jordan, a study by Abuhammad et al. (8) surveyed 447 Jordanian nurses to examine the barriers they faced with respect to participating in research. The findings indicated that over half of the respondents perceived several significant obstacles, particularly those related to organizational factors and related to individual characteristics such as age, experience, and nationality. Similarly, AbuRuz et al. (10) found that female nurses were less likely to engage in research activities, demonstrating both less favorable attitudes and lower levels of research knowledge than their male counterparts. Nurses holding a master’s degree (MSc), those working in intensive care units (ICUs), and those employed in private hospitals were reported to have more positive attitudes toward research and higher levels of knowledge and skills than nurses with only a bachelor’s degree (10). Da’seh and Rababa (28), however, highlighted that work environment, and particularly lack of time due to heavy workloads, could act as a significant barrier to research utilization, especially in public hospitals in Jordan.

Jordan is a Middle Eastern country recognized for its proficiency in the healthcare sector. The nursing sector in Jordan has thus made considerable progress in both clinical practice and research development over the years. Nevertheless, the quantity of studies conducted is restricted, predominantly executed by academics, with only a handful of such investigations involving Jordanian nurses employed as registered nurses in hospitals (11). Numerous studies have also investigated the perceived obstacles encountered by Jordanian nurses in terms of applying research findings (8, 11, 28), as well as their attitudes towards evidence-based practice (10). Additionally, investigation has been undertaken regarding the views of nursing students towards research in nursing (12). However, to the best of the current researchers’ knowledge, no research to date has explicitly examined how Jordanian nurses feel about doing nursing research. Moreover, scant and frequently contradictory information exists concerning the correlation between various demographic variables in the nursing population, such as age, gender, job role, experience, and educational attainment, and their attitudes toward engaging in nursing research. While existing literature has documented nursing research attitudes in various countries, limited evidence exists regarding nurses’ attitudes and perceived barriers in Jordan. This study aims to contribute new insights to this underresearched context. The aim of this study was therefore to examine more specifically the attitudes of Jordanian nurses regarding the conduct of nursing research. The study’s objectives were thus

1. To explore the perspectives of Jordanian nurses towards the conduction of nursing research.
2. To investigate differences in Jordanian nurses’ attitudes toward conducting nursing research based on sociodemographic variables.

## Materials and methods

### Design and sample

This study utilized a cross-sectional descriptive design to explore the attitudes of Jordanian nurses toward the conduction of nursing research. This study used a convenience sample of Jordanian registered nurses employed across various private and governmental sectors. Participants were invited to participate by means of a cover letter that required them to respond “I agree” to a statement regarding the survey procedures and content to proceed to the completion of the survey. A registered nurse was defined as a practitioner with a bachelor’s degree in nursing or higher. The target population was thus comprised of all registered nurses in Jordan employed across various sectors, whereas the exclusion criteria pertained to non-Jordanian nurses and currently unemployed individuals. G. Power 3.1.9.7 software was used to determine the required sample size. *T*-tests and ANOVA analysis were used to explore differences in nurses’ attitudes towards the conduction of nursing research based on the following demographic data: age, gender, education, marital status, years of experience, involvement/expertise in research, enrollment in research courses, responsibility of role, nursing specialty, and ward type. A minimum sample size of 156 participants, based on a medium effect size, power = 0.80, and *α* = 0.05, was required to conduct ANOVA analysis; however, the sample size was increased to 220 to reduce any effects from incomplete questionnaires or a significant nonresponse rate.

### Setting of the study

The Royal Medical Services (RMS), which is affiliated with Jordan’s Armed Forces, includes King Hussein Medical City in Amman, a major referral center with 1,414 beds spread across five hospitals, including general, cardiac, pediatric, rehabilitation, transplant, and laboratory/research facilities. RMS provides comprehensive tertiary care for military personnel, families, and civilians, including emergency, ICU, surgical, and transplant services.

The Ministry of Health runs 31 public hospitals with a total of 5,884 beds, which accounts for approximately 35% of Jordan’s total hospital capacity. These hospitals provide secondary and tertiary care, which includes internal medicine, surgery, pediatrics, maternity care, emergency services, and specialty clinics.

Jordan’s private healthcare sector includes hospitals with approximately 5,529 beds, accounting for 36% of the country’s hospital beds. These hospitals range from general to specialized facilities.

Jordan University Hospitals are two; one is located in Amman and affiliated with the University of Jordan, and the second is King Abdallah Hospital in Irbid City and affiliated with Jordan University of Science and Technology. Both are referral and teaching hospitals.

### Instruments

Data were gathered via the Boothe Attitudes Toward Nursing Research instrument. The scale was created by Boothe in 1981 and subsequently modified by Bostrom and associates in 1989. The questionnaire consists of two sections. The first section includes 46 items, broken down into three subscales, intended to evaluate nurses’ attitudes toward nursing research. The subscales include (a) interest and environmental support (21 items), (b) payoff and benefits (17 items), and (c) barriers to conducting research (eight items). Authorization to utilize the modified scale was acquired from Bostrom by means of email correspondence. The survey questionnaire and forms were developed and used in the English language.

The reliability of the original scale reported by Boothe for both the full scale and the subscales ranges from 0.65 to 0.91 (29). The coefficient alpha for the modified scale, as reported by Bostrom, ranges from 0.64 to 0.87 (30). As the instrument’s item responses are organized from strongly disagree at a score of 1 to strongly agree at 5, the aggregate score could vary from 46 to 230. A high score on positive items indicates more favorable attitudes toward research, while a high score on negative items indicates more unfavorable attitudes toward research.

The second section sought demographic data from participants, including age, gender, marital status, educational attainment, years of experience, motivation for pursuing continuing nursing education, role responsibilities, nursing specialty, research participation experience, enrollment in research courses, and ward assignments.

In this study, five-point Likert scale data was considered an interval scale and interpreted based on mean range. The minimum and the maximum length of the 5-point Likert-type scale mean that the range is calculated as 5 − 1 = 4, which is then divided by five as the greatest value within the scale (4 ÷ 5 = 0.80) (13, 14). The number one, which is the lowest possible value in the scale, is then added to identify the maximum of each cell. The length of the cells can then be determined and interpreted as follows: From 1 to 1.80 represents an average response of 1, strongly disagree; from 1.81 to 2.60 represents 2, disagree; from 2.61 to 3.40 represents 3, uncertain; from 3.41 to 4.20 represents 4, agree; and from 4.21 to 5.00 represents 5, strongly agree.

### Ethical considerations

This research was performed in compliance with the ethical principles established in the Declaration of Helsinki. The Institutional Review Board (IRB) at Mutah University in Al-Karak, Jordan, granted approval to conduct the study among Jordanian nurses employed in private and public sectors. The researchers gathered data through an electronically administered self-completed questionnaire posted on WhatsApp and sent via direct e-mails and the Facebook platform. An invitation outlining the study’s goal, stressing that participation would be both anonymous and voluntary, was included in the cover letter sent to all participants.

The paper was called a “cover letter,” but it was essentially an informed consent form for giving approval. The cover letter informed participants that they could withdraw from the study without penalty or compensation and that their information would be kept confidential and utilized exclusively for research purposes. The questionnaire also began with a statement requesting participant consent, confirming that all electronic data will be retained on the primary researcher’s computer for 5 years. We obtained authorization to use the research instrument in this study by electronically contacting the original researcher.

### Data collection

Data was gathered from Jordanian nurses working in the public and private sectors, with Google Forms used to gather responses from an electronic self-administered survey over the period of October 2024 to November 2024. The questionnaire was disseminated via multiple platforms, including Facebook, e-mail, and WhatsApp. A cover letter was used to convey essential details about the study’s goal, possible risks, and the procedures used to collect data to participants. Additionally, the principal investigator’s contact information (email and phone number) was provided in case of any inquiries or concerns.

### Data analysis

The primary variables were analyzed using IBM SPSS Statistics version 28.0. All data were first examined for missing values and entry errors and cleaned accordingly. Categorical variables were summarized using frequencies and percentages, while continuous variables were described using measures of central tendency (mean and median) and dispersion (standard deviation).

Independent samples *t*-tests were used to examine differences in mean scores between two independent groups (e.g., gender, further education). One-way ANOVA was employed to assess differences in attitudes across multiple groups (e.g., years of experience). When significant differences were found, Tukey’s *post-hoc* test was conducted to identify specific group differences. These tests were selected because the assumptions for regression analysis were not met. A *p*-value less than 0.05 was considered statistically significant.

## Findings

### Sample characteristics

The study sample consisted of 220 registered nurses from Jordan, employed across various healthcare sectors, including the Ministry of Health (MOH), Royal Medical Services (RMS), university hospitals, Jordanian universities, and the private health sector. Most participants were aged between 20 and 40 years (62.7%, *n* = 138), and 55.5% were male (*n* = 122). Most participants were married (79.5%, *n* = 175), while the remaining 20.5% were single, divorced, or widowed. Approximately 42.7% (*n* = 94) of the sample were employed in public hospitals (MOH and RMS). (See Table 1 for further details).

**Table 1:** Demographic characteristics of participants (*n* = 220).

Characteristic	Frequency (*n*)	Percentage (%)
Age (years)		
20–30	35	15.9
31–40	103	46.8
41–50	48	21.8
51–60	29	13.2
>60	5	2.3
Gender		
Male	122	55.5
Female	98	44.5
Marital status		
Single	41	18.6
Married	175	79.5
Divorced	3	1.4
Widowed	1	0.5
Place of employment		
MOH	52	23.6
RMS	42	19.1
University hospitals	75	34.1
Jordanian Universities	32	14.5
Private hospitals	19	8.6
Experience		
0–20	91	41.4
21–30	90	40.9
>30	39	17.7
Job responsibilities		
Staff nurse	78	35.5
Charge nurse	47	21.4
Unit coordinator	25	11.4
Manager/director	20	9.1
Educator	50	22.7
Have you received further research education		
Yes	116	52.7
No	104	47.3
When did you take the research course		
Baccalaureate degree	152	69.1
Master’s degree	33	15.0
PhD degree	14	6.4
In-service education	5	2.3
Did not take any course	16	7.3
Nature of employment		
Academic nursing	32	14.5
Clinical nursing	188	85.5
Participation in research activity		
Yes	95	43.2
No	125	56.8

### Attitudes of nurses towards the conduction of nursing research

The study used descriptive statistics for all items of the Boothe Attitudes Scale and for the three subscales to identify attitudes of Jordanian nurses towards conducting nursing research. The overall mean score of the Boothe subscales was 3.289, SD = 0.968, which indicates uncertainty with respect to attitudes toward research, with the mean lying within the range of 2.61–3.40. Particularly with respect to the first subscale, “Interest and environmental support,” the reported attitudes toward research indicated strong uncertainty or ambivalence (mean = 3.20, SD = 0.979), while in the second subscale, “Payoff and benefits,” attitudes toward research were more positive (mean = 3.439, SD = 1.038), as this lies within the range of 3.41–4.20. The mean score for the third subscale, “barriers to conducting research,” had a mean of 3.201, SD = 0.988, again indicating uncertainty, as illustrated in Table 2.

**Table 2:** Mean scores of the subscales of the Boothe Attitudes towards Nursing Research Scale (*n* = 220).

Scale and subscale	Mean ± SD	Interpretation
Boothe Attitude scale	3.29 ± 0.97	Uncertain
Interest and environmental support subscale	3.20 ± 0.98	Uncertain
Payoff and benefits	3.44 ± 1.04	Agree
Barriers to conducting research	3.20 ± 0.99	Uncertain

The overall means for the Boothe’s Attitudes Toward Nursing Research Scale ranged from 50 to 207, with an arithmetic mean of 150 (SD = 42). The highest mean was found in the interest and environmental support subscale, with an arithmetic mean of 63 (SD = 18). With respect to the Payoff and Benefits subscale, the results ranged from 18 to 84, with an arithmetic mean of 58 (SD = 17). The mean results on the third subscale (Barriers to Conducting Research) ranged from 9 to 36, with an arithmetic mean of 25 (SD = 7.0), as shown in Table 3.

**Table 3:** Means of the total score of subscale attitudes of nurses toward nursing research (*n* = 220).

Scale and subscale	Min	Max	Mean ± SD	P25%	P50%	P75%
Interest and environmental support subscale	22	87	63 ± 18	49	68	78
Payoff and benefits	18	84	58 ± 17	51	65	69
Barriers to conducting research	9	36	25 ± 7	21	27	31
Boothe Attitude scale	50	207	150 ± 42	132	163	182

Descriptive statistics were used to determine how Jordanian nurses felt about each item on the Boothe attitudes scale, based on finding the mean and the standard deviation (SD). Each item was ranked in descending order based on the mean score, from the highest mean score, which indicated a higher level of agreement, to the lowest mean score, which indicated a higher level of disagreement with the item statement; overall, the mean score was assumed to represent participant agreement with, uncertainty around, or disagreement with a given statement.

This study showed that 15 items out of the 46 items on the Boothe scale achieved the highest levels of agreement, suggesting that respondents felt positively about those aspects of conducting nursing research. These 15 items appeared across the first subscale, “Interest and environmental support,” the second subscale, “Payoff and benefits,” and the third subscale, “Barriers to conducting research.” As perceived by Jordanian nurses, the three most positive items in the first subscale, “Interest and environmental support,” were “I am interested in conducting research,” “I would like to conduct research,” and “I would like to put research high on my list of priorities.” Eleven of the most agreeable items related to the second subscale “Payoff and benefits”: “I would conduct research if I had the time,” “I would conduct research if I knew how to write the proposal, conduct and analyze the results and findings,” “Nurses would conduct more research if more funds were available for them to use for this purpose,” “Members of the treatment team other than nurses should conduct research relative to patient care,” “Nursing research is conducted because it allows nurses to be promoted,” “Nursing research is the means whereby the theoretical basis for nursing practice is derived, “I would do research if I knew more about it,” “I would like to conduct a study of a problem in patient care,” “I believe that I would conduct research if someone more knowledgeable would help me in the process,” “Nurses would conduct research if relief time were given to conduct research,” and “Nurses would conduct research if they were provided time for research.” The only item from the third subscale, “barriers to conducting research,” within this category was “I have the skills and knowledge necessary for me to conduct research.”

On the other hand, the lowest mean scores, which represent a higher level of disagreement with the item statement, were seen for two items from the first subscale and one from the third subscale. The items from the first subscale in this category were “I believe my place of employment has ample secretarial assistance for anyone wishing to conduct research” and “I believe my job provides the time necessary to conduct research,” while the relevant item from the third subscale was “Patient participation in nursing research is difficult to obtain” (Table 4).

**Table 4:** Mean scores on the Boothe Attitudes scale ranked in descending order (*n* = 220).

#	Item	Mean ± SD	Scale
22	I would conduct research if I had the time	3.63 ± 1.19	II
23	I would conduct research if I knew how to write the proposal, conduct and analyze the results and findings	3.62 ± 1.24	II
34	Nurses would conduct more research if more funds were available for them to use for this purpose	3.60 ± 1.23	II
26	Members of the treatment team other than nurses should conduct research relative to patient care	3.57 ± 1.19	II
3	Nursing research is conducted because it allows nurses to be promoted	3.56 ± 1.32	II
36	I am interested in conducting research.	3.55 ± 1.23	I
25	Nursing research is the means whereby the theoretical basis for nursing practice is derived	3.52 ± 1.14	II
1	I would like to conduct research	3.52 ± 1.37	I
2	I would like to put research high on my list of priorities.	3.50 ± 1.31	I
41	I would do research if I knew more about it.	3.49 ± 1.20	II
29	I would like to conduct a study of a problem in patient care	3.48 ± 1.16	II
44	I believe that 1 would conduct research if someone more knowledgeable would help me in the process.	3.46 ± 1.23	II
39	Nurses would conduct research if relief time were given to conduct research.	3.45 ± 1.16	II
17	I have the skills and knowledge necessary for me to conduct research	3.41 ± 1.19	III
38	Nurses would conduct research if they were provided time for research	3.41 ± 1.20	II
30	I would conduct research if patient assignments were lightened	3.40 ± 1.20	II
24	Research findings that are advantageous to good patient care can be implemented in my working environment.	3.38 ± 1.18	II
32	Nursing research should be initiated by nurses in the clinical area	3.37 ± 1.26	II
33	Nursing research should be initiated by nurses in education	3.37 ± 1.24	III
45	Nursing research should be initiated by nurse researchers	3.35 ± 1.26	I
8	I know what is expected of me when submitting my research proposal to the hospital nursing research committee.	3.32 ± 1.17	I
27	Nursing research requires more from me than I am willing to give to my job.	3.32 ± 1.21	I
7	The process of submission of the research proposal to the hospital nursing research committee is too detailed.	3.31 ± 1.22	III
28	Nursing research should be conducted by nurses with a baccalaureate degree.	3.29 ± 1.22	III
11	I am familiar with selected statistical procedures used for the analysis of research findings.	3.23 ± 1.22	I
15	I believe my peers in nursing would assist in conducting research.	3.22 ± 1.18	I
4	I believe my place of employment would provide me with ample assistance during the research process.	3.22 ± 1.28	I
37	Nurses receive praise from their peers and colleagues when they conduct research.	3.20 ± 1.23	II
5	I believe my place of employment would provide me with ample consultive assistance for conducting research.	3.20 ± 1.27	I
14	My peers in nursing would encourage conducting research.	3.19 ± 1.21	I
16	My job provides ongoing educational programs to conduct research.	3.18 ± 1.26	I
43	Nursing research should be conducted by nurses with a master’s degree.	3.16 ± 1.22	III
13	My colleagues (other professionals) would encourage me to conduct research.	3.13 ± 1.25	I
35	Time spent giving patient care is more important than time spent conducting research.	3.12 ± 1.29	I
10	The informed consent necessary for patient participation prevents me from conducting research in my work areas.	3.12 ± 1.27	III
18	I believe my working environment provides ample opportunity to conduct research.	3.11 ± 1.23	I
21	I believe my place of employment has ample assistance for anyone for the analysis of results and findings of the research that is conducted.	3.09 ± 1.22	I
42	Nurses are criticized too much by their peers when they conduct research.	3.08 ± 1.22	II
20	I believe my place of employment has ample statistical assistance for anyone wishing to conduct research.	3.06 ± 1.32	I
9	The informed consent necessary for employee participation in research prevents me from conducting research in my work areas.	3.05 ± 1.30	II
40	Nursing research should be conducted by nurses with a doctorate.	3.02 ± 1.34	III
6	My supervisor would allow time in my daily assignment to conduct research.	3.01 ± 1.23	I
31	Nursing research is more essential in the medical setting than in the psychiatric setting.	2.77 ± 1.14	I
19	I believe my place of employment has ample secretarial assistance for anyone wishing to conduct research.	2.51 ± 1.17	I
12	1 believe my job provides the time necessary to conduct research.	2.48 ± 1.18	I
46	Patient participation in nursing research is difficult to obtain.	2.46 ± 1.15	III

#, The order number of the item in the original scale. Scale (I), Interest and Environmental Scale. Scale (II), Two-Payoff and benefits Scale. Scale (III), Barriers to conducting research.

### Factors affecting the attitudes of nurses toward conducting research

Analysis of variance (ANOVA) was used to determine whether there was any significant difference between the group means based on demographic and similar factors. This process was applied due to the fact that the assumptions required for regression analysis were not met. The results showed a statistically significant difference in how people across the five identified age groups felt about nursing research (*p* < 0.001). The age groups used were 20–30, 31–40, 41–50, 51–60, and over 60 years old. *Post hoc* analysis using the Bonferroni test indicated that participants aged 20–30 offered significantly higher scores compared to other age groups. Similarly, there was a statistically significant difference regarding how people felt about research based on their jobs, with a contrast between clinical and academic settings (*p* < 0.001). In particular, teachers said they were more committed to research than other groups. Furthermore, the findings also revealed a statistically significant relationship between the timing of having undertaken a research course and attitudes toward nursing research (*p* = 0.01). Post hoc analysis indicated that participants who took such a course during PhD studies demonstrated a higher affinity for research as compared to those who completed such courses at more basic educational levels. The analysis also revealed a statistically significant effect from years of experience regarding attitudes toward nursing research (*p* < 0.001). *Post hoc* pairwise comparisons indicated that participants with 0–20 years of experience reported significantly higher mean scores than those with more than 20 years of experience. As Table 5 shows, however, there was no statistically significant difference in how nurses felt about doing nursing research based on their marital status (*p* = 0.69) or where they worked (*p* = 0.73).

**Table 5:** Analysis of variance (One way ANOVA) of attitudes toward nursing research (*n* = 220).

Variables	Category	Frequency	Mean ± SD	*F*-value	*p*-Value
Age groups (years)	20–30	35	180.6 ± 18.05	23.05	<0.001
	31–40	103	160.4 ± 32.85		
	41–50	48	125.2 ± 44.31		
	51–60	29	111.7 ± 45.51		
	>60	5	159.4 ± 10.78		
Marital status	Single	41	155.9 ± 45.47	0.49	0.69
	Married	175	147.8 ± 41.71		
	Divorced	3	160.3 ± 15.04		
	Widowed	1	–		
Nurses place of employment	MOH	75	149.69 ± 40.7	0.51	0.732
	RMS	52	147.19 ± 47.66		
	University hospitals	42	148.64 ± 41.24		
	Jordanian hospitals	32	158.13 ± 28.64		
	Private hospitals	19	142.53 ± 53.24		
Years of experience	0–20	91	164.68 ± 31.5	31.08	<0.001
	21–30	90	151.87 ± 38.9		
	>30	39	108.67 ± 45.3		
Job responsibilities	Staff nurse	78	169.38 ± 26.79	25.88	<0.001
	Charge nurse	47	139.6 8 ± 44.99		
	Unit coordinator	25	120.12 ± 46.90		
	Manager/director	20	95.35 ± 41.50		
	Educator	50	164.10 ± 24.05		
When did they take a research course?	Baccalaureate degree	152	142.78 ± 47.37	3.39	0.01
	Master’s degree	33	161.36 ± 22.33		
	PhD degree	14	169.93 ± 21.33		
	In-service education	5	167.20 ± 14.96		
	did not take any course	16	165.56 ± 16.06		

SD not calculated for groups with n = 1.

*T*-tests were used to assess whether there was any statistically significant difference between groups based on demographic variables. The findings showed no statistically significant difference between the male and female groups in terms of attitudes towards the conduction of nursing research, *F* (2,218) = 3.512, *p* = 0.062. However, the results did reveal that changes in several other variables led to statistically significant differences in nurses’ attitudes towards conducting nursing research: these were obtaining further research education (*F* (2, 218) = 39.993, *p* = 0.000), engagement in conducting research (*F* (2, 218) = 26.251, *p* = 0.000), and receiving an incentive for conducting research (see Table 6).

**Table 6:** Mean difference in levels of attitude towards conducting nursing research based on nurses’ demographics (*n* = 220).

Variables	Categories	Frequency	Mean ± SD	*T* value	*p*-Value
1. Gender	Female	122	146.8 ± 44.2	−0.540	0.062
	Male	89	152.7 ± 39.2		
2. Further education	Yes	116	158.7 ± 32.7	3.536	0.000
	No	104	139.2 ± 48.7		
3. Participant engagement in conducting research	Yes	95	157.1 ± 32.6	2.346	0.000
	No	125	143.8 ± 47.5		
4. Incentive	Yes	187	149.8 ± 43.6	0.214	0.048
	No	33	148.1 ± 33.2		

## Discussion

The purpose of the current study was to examine nurses’ attitudes toward conducting nursing research. The results were based on analyzing information gathered from 220 Jordanian nurses working in various fields, such as fieldwork, academia, the government, and the private sector. The questionnaires featured 46 items across three topics: interest and environmental support, payoff and benefits, and barriers to conducting research. The findings indicated, overall, that nurses exhibit a distinct sense of uncertainty regarding their attitudes toward research.

The average score of the Boothe subscales was 3.289, with a standard deviation of 0.968. This outcome offers a contrast to the conclusions of an integrative review of 236 articles that revealed nursing students generally possess positive attitudes toward research and recognize its value in practice (15). Although the findings are not in contact with a study involving 132 nurses, who similarly exhibited positive attitudes. The discrepancy may be attributed to the small sample size and different clinical area (7). However, they are consistent with the findings of Kovačević et al. (16), who showed that nurses had only mildly positive attitudes toward conducting nursing research, crucial for promoting evidence-based practice in healthcare. A study found a strong link between reading academic articles and positive research attitudes among nurses. Institutions should foster research by establishing dedicated nursing research centers and supporting continuing education. Nurse managers must address barriers to participation, enhance research attitudes, and implement strategies for increased engagement and evaluation of research involvement to create a supportive research environment (17). When examining the Boothe’s Attitudes to Nursing Research Scale, the “Interest and Environmental Support” and “Barriers to Conducting Research” subscales suggest that nurses need more help. The least amount of satisfaction was seen in terms of getting written help and having enough time for research, which is in line with Nkrumah et al. (18) and Roxburgh (19). Scala et al. (20) reported that lack of expertise, time, and support are all mentioned as recurring hurdles to nurses doing research. This indicates the need for training programs for nurses focused on research writing, as well as the development of ways to offer them more time to engage in research activities.

Nurses generally showed more positive attitudes (mean = 3.439) across the “Payoff and Benefits” subscale, expressing strong interests in pursuing research where they had more free time, proposal writing help, and adequate funding. This aligns with prior findings that such facilitators are crucial for developing evidence-based practice (21, 22). In the same detection, Chen et al. (23) identify barriers to nurses’ study engagement as unclear topics, insufficient instruments and skills, and contextual factors.

Further analysis of nurses’ attitudes toward research revealed differences based on educational levels and age. As with other studies (24), nurses with higher levels of education scored the Boothe’s Attitudes to Nursing Research instrument more positively. This is likely to be because nurses with more education tend to have better attitudes towards research, as advanced education familiarizes nurses with the research process. Uysal Toraman et al. (31) confirmed that nursing students in Turkey who wrote master’s theses on scientific research displayed improved attitudes and knowledge regarding research. Additionally, Hagedoorn et al. (25) demonstrated distinctions between BSNs and diploma-level nurses, with more highly educated nurses viewing research as an essential part of their role and thus feeling better prepared to undertake it. Enhanced educational levels and training are therefore key to encouraging positive research attitudes. Hospital workers also expressed the view that research is important to support in-patient care; however, their priority is to provide services to patients, consistent with a study by Westlake et al. (26) that found that heart failure nurses with higher education levels showed increased confidence, involvement, and interest in research. This serves as a crucial reminder that nurses must be encouraged to improve their practice by fortifying the connection between patient care and research findings, thereby enhancing quality of care.

The current study revealed significant age group differences regarding attitudes toward nursing research, with respondents aged 20 to 30 displaying more positive attitudes than older individuals. This finding supports Vijayalakshmi et al. (7), who indicated that younger nurses excel in terms of research participation and attitudes. This study also confirms that younger nurses show greater enthusiasm for research as compared to their middle-aged and older counterparts, potentially highlighting a correlation with motivation to undertake ongoing professional education. In contrast, a study conducted by Devrani et al. (3) did not identify differences in attitudes among nurses based on varying educational levels, instead suggesting that nurses with more than 20 years of experience were the least interested in research. Overall, nurses working in the academic field exhibited increased interest and participation in research as compared to those working in practical environments such as hospitals, due to the presence of additional support and incentives. Furthermore, their greater knowledge of writing research papers may support their enthusiasm, in alignment with a larger study, which found that only 11% of nurses actively participate in writing scientific papers, which points to the need for increased research participation. Policymakers seeking to enhance nursing research should thus seek to provide protected time, education, collaboration, and financial support, while hospital managers should foster a research culture to enhance competencies and evidence-based practice (27). Several international studies have demonstrated that structured interventions—such as research mentorship programs, provision of protected research time, and institutional recognition of research engagement—can significantly enhance nurses’ participation in research activities (4, 19).

The current study’s findings, while indicating unclear attitudes toward research among Jordanian nurses, are not universally applicable to all nurses. Uncertain attitudes would also not preclude engagement in research. Figuring out how nurses feel is only the first step, and future researchers must therefore work to link these feelings to other important factors to understand how nurses’ feelings about changing their practices and implementing evidence-based healthcare may impact both the performance and implementation of research.

### Recommendations

The findings of this study indicate several effective strategies to enhance nurse participation in research. Enhancing nurses’ practical training, particularly in areas such as composing research proposals and data analysis, improves their confidence and competence in conducting studies. It is essential to allocate time for nurses to engage in research within their demanding schedules. When nurses receive time off for research and their institutions provide support and encouragement, they are more inclined to engage in such activities. Minor factors such as receiving mentorship, acknowledgment, or simply recognizing the significance of their contributions can substantially impact individuals. Ultimately, integrating research as a fundamental component of professional advancement rather than a supplement can transform its practical implementation. When research is integrated into a nurse’s professional practice and supported by the surrounding system, it enhances the nurse’s development and improves the quality of care for all patients.

### Clinical implications

Hospital administrators may seek to enhance nurses’ attitudes toward conducting nursing research by implementing policies such as a clinical ladder, incentives, and targeted support and funding to promote the development of high-quality nursing research. Additionally, the establishment of a center for research that can guide and assist inexperienced researchers in developing and coming up with creative solutions to clinical problems may also improve standards of care. Policies of this kind should emphasize research time, ongoing education, and external motivators to encourage nurses to engage in research. Additionally, hospital administrators may seek to collaborate with academic institutions to improve their staff members’ knowledge and mindsets regarding nursing research. Based on this, hospital managers may also seek to provide research courses, workshops, training sessions, seminars, and scientific days to enhance both positive attitudes toward and the execution of nursing research. It is recommended that nursing managers and educators in Jordan consider providing targeted training in research methods and data analysis, as well as creating institutional policies that allocate protected time for staff nurses to engage in research activities.

### Limitations

This study contributes to existing knowledge regarding attitudes of Jordanian nurses toward nursing research; however, its results indicate a requirement to examine the impact of specific interventions on the quantity and quality of nursing studies produced. Additionally, since the data relied on self-reported responses, it is important to take response bias into account when interpreting the findings.

## Conclusion

The current study found that Jordanian nurses demonstrate uncertainty regarding their attitudes toward the conduction of nursing research. Consequently, hospital administrators may wish to establish workshop training courses, seminars, and conferences to improve research acceptance and execution. Nurses in the study exhibited favorable attitudes towards the rewards and advantages of engaging in nursing research, with the highest mean agreement observed in terms of interest and environmental support. Consequently, hospital administrators may seek to allocate funds to facilitate research and provide incentives for exemplary studies, as well as provide protected time and leave for dedicated nurse researchers.

This study also demonstrated that education and involvement in research activities influence nurses’ attitudes toward conducting nursing research. As a result, hospital administrators should also seek to promote and facilitate the attainment of higher education among nurses in order to enhance patient outcomes and ensure that nursing practices are safe and evidence-based.

## Data Availability

The raw data supporting the conclusions of this article will be made available by the authors, without undue reservation.
